# Compressing Proteomes: The Relevance of Medium Range Correlations

**DOI:** 10.1155/2007/60723

**Published:** 2007-10-30

**Authors:** Dario Benedetto, Emanuele Caglioti, Claudia Chica

**Affiliations:** 1 Dipartimento di Matematica, Università di Roma "La Sapienza", Piazzale Aldo Moro 5, Roma 00185, Italy; 2 Structural and Computational Biology Unit, EMBL Heidelberg, Meyerhofstraße 1, Heidelberg 69117, Germany

## Abstract

We study the nonrandomness of proteome sequences by analysing the correlations that arise between amino acids at a short and medium range, more specifically, between amino acids located 10 or 100 residues apart; respectively. We show that statistical models that consider these two types of correlation are more likely to seize the information contained in protein sequences and thus achieve good compression rates. Finally, we propose that the cause for this redundancy is related to the evolutionary origin of proteomes and protein sequences.

## 

[1,2,3,4,5,6,7,8,9,10,11,12,13,14,15,16,17,18,19,20,21,22,23,24,25,26,27,28,29,30,31]
